# Characterisation of UHMWPE Polymer Powder for Laser Sintering

**DOI:** 10.3390/ma12213496

**Published:** 2019-10-25

**Authors:** Yas Khalil, Neil Hopkinson, Adam Kowalski, John Patrick Anthony Fairclough

**Affiliations:** 1 Department of Mechanical Engineering, University of Sheffield, Sheffield S3 7HQ, UK; 2 Xaar 3D Ltd, 5-6 William Lee Buildings, Science Park, Nottingham NG7 2RQ, UK; 3 Unilever plc, R & D Port Sunlight Laboratory, Wirral CH63 3JW, UK

**Keywords:** UHMWPE, Laser sintering, powder flow, hot stage, crystallinity

## Abstract

Ultra-high molecular weight polyethylene (UHMWPE) is a thermoplastic semicrystalline polymer that has outstanding mechanical properties, low friction coefficient, excellent wear resistance, and is highly resistant to corrosive chemicals. UHMWPE is found in many applications including artificial joints and filtration. However, UHMWPE parts cannot be produced easily by traditional techniques, such as injection moulding and extrusion because of its very high melt viscosity owing to the extremely long polymer chains. Few attempts were made to process UHMWPE by additive manufacturing, particularly laser sintering. This is due to the lack of understanding of the powder properties of UHMWPE. Therefore, the aim of the powder characterisation process in this study is to gain a better understanding of the material requirements and provide a detailed insight on whether UHMWPE is a suitable material for laser sintering. The characterisation process includes powder morphological and flow characteristics, thermal behaviour and stability, and crystallinity of UHMWPE. The study reveals that the sintering behaviour of polymers is controlled by the morphology of the particles in addition to the viscous flow of UHMWPE. There are still difficulties of processing UHMWPE due to highly agglomerated structure of smaller particles with the presence of fibrils in the UHMWPE particles.

## 1. Introduction

Powder characterisation is an essential step for processing polymers by laser sintering (LS). Laser sintering is well suited process to manufacture a variety of polymeric materials including non-standard laser sintering materials (i.e., off-the-shelf powders). However, powder characteristics and properties, such as particle size, shape and powder flow, can greatly influence the quality of the parts produced as well as the processing of the powder [1]. However, no single analytical method alone can be used to reliably characterise a powder. Methods such as x-ray powder diffraction, scanning electron microscopy, density, thermal analysis, hot stage microscopy, and X-ray diffraction (XRD) analysis all contribute to define the powder characteristics that best suited for laser sintering.

Previous investigations show that the degree of control over the microstructure of the laser sintered parts depends on the process parameters, especially the properties of the powder. Particle size distribution and morphology influence the packing density of the powder, whereas the thermal behaviour and stability define the laser energy density required in laser sintering [2]. Understanding the thermal stability of ultra-high molecular weight polyethylene (UHMWPE) powder is essential for the simulation of laser sintering process.

Salmoria et al. [3] successfully processed high density polyethylene (HDPE) by laser sintering. They were able to show that the structure and the properties of the laser-sintered parts can be controlled by using different particle sizes. Their results showed that a lower sintering degree was achieved with larger particle size with lower values for the mechanical properties. Smaller particles form a larger contact area, contributing to a higher rate of necking between particles and resulting in improved mechanical properties [4].

Few attempts, however, have been made to process UHMWPE by laser sintering. Rimell and Marquis attempted to produce parts for clinical application from UHMWPE (GUR 4170 and GUR 4120). A bespoke laser sintering machine was used in their trials. However, the attempts to produce multilayer parts were unsuccessful. Many issues were developed during laser sintering, including a high degree of shrinkage and curling [5]. Goodridge et al. produced LS parts from UHMWPE (GUR 4170) with a high sintering between particles and layers. A commercial Vanguard Laser Sintering machine from 3D Systems was used in their study [6]. Goodridge et al. performed a number of trials to establish suitable parameters for processing UHMWPE. Different laser powers, bed and feed temperatures and scan counts were used. They indicated that the processing window to obtain UHMWPE components was narrow and therefore precise process parameters (i.e., laser power combined with correct temperature for the bed and the feed) were required. Goodridge et al. reported that the UHMWPE laser sintered parts were produced by using a laser power of 13 watts, double scan count with bed and feed temperatures of 135 °C and 125 °C, respectively. The mechanical properties of the sintered parts were investigated by performing tensile and three-point bend tests. The results show that the ultimate tensile strength and Young’s modulus were just above 0.2 MPa and 1.5 GPa, respectively and the flexural strength and modulus were 0.52 ± 0.2 MPa and 18.67 ± 4.3 MPa, respectively.

The aim of this work is to obtain a better understanding of the material requirements and provide a detailed insight on whether UHMWPE GUR 2122 is a suitable material for laser sintering.

### 1.1. Polymer Sintering Model

Sintering models have been used as a means to investigate the suitability of materials for sintering. These models are based on two symmetric spherical or cylindrical particles in contact with each other and are concerned with the neck growth occurs during sintering. This behaviour can be verified by observing the consolidation optically using hot stage microscopy [7].

**Figure 1:**
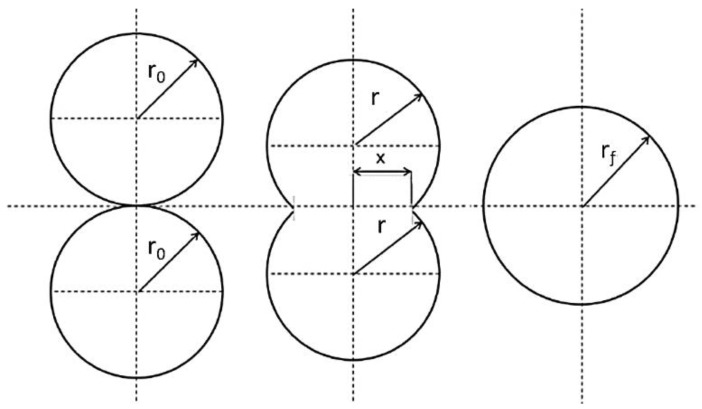
Schematic of sintering of two spherical particles. Redraw from reference [7].

Frenkel’s model is one of the early theories used to describe the principle of sintering [8]. However, it has since undergone many modifications to remove some of the approximations associated with this theory and also to allow application to individual situations. Frenkel’s analytical model describes the rate of coalescence of two identical spherical particles (Figure 1) via the viscosity of the material and the surface tension over a given time period at elevated temperatures. The model suggests that the rate of sintering is inversely proportional to the melt viscosity of the polymer at low shear rate [7].

Frenkel’s model is described by the following Equation (1):

(1) $$\frac{x}{r}\;=\;{\left(\frac{3\gamma \times t}{2\eta \times r_{0}}\right)}^{\frac{1}{2}}$$

where *x*, *r*_0_, *r*, *γ*, *t*, and *η* are sintering neck radius, initial particle radius, particle radius, surface tension, sintering time, and zero-shear viscosity of the polymer, respectively. Frenkel’s model is restricted to Newtonian flow, where viscosity remains constant, and is only valid for the initial stages of sintering, when the diameter of the particle remains relatively fixed. It was noticed that the viscosity used in Frenkel’s model is three folds higher than the zero shear viscosity and therefore this model was modified and subsequently became known as Frenkel/Eshelby model [9] Equation (2).

(2) $$\frac{x}{r}\;=\;{\left(\frac{\gamma \times t}{\eta \times r_{0}}\right)}^{\frac{1}{2}}$$

These models are difficult to apply to irregularly shaped particles due to poorly defined dimensions and necking of these particles [10]. The sintering behaviour of polymers is controlled by the morphology of the particles in addition to the factors included in the Frenkel-Eshelby model [10]. However, the model provides insight into the influence of material properties and particle size on the coalescence rate and it has been used by many authors as part of a materials characterisation process to investigate sintering behaviour [7,11,12,13,14,15,16].

### 1.2. Thermal Transition

The glass transition, melt, and flow temperature are the major thermal transitions that many polymers go through. The glass transition of UHMWPE occurs around −160 °C. The smaller lamellae in UHMWPE begin to melt when temperature rises above 60–90 °C [17]. The melt temperature may occur between 112 °C and 150 °C and crystallisation during cooling between 85 °C and 112 °C [6]. If the melting and crystallisation peaks are very close or overlapped, the processed material will quickly crystallise as soon as it cools, resulting in shrinkage and deformation. Therefore, polymers that can be processed at a wide range of temperatures are the more desirable in laser sintering. These materials allow a better processing freedom and space, especially when optimising processing parameters [6]. Additionally, the literature indicates that the values of the glass transition and melting temperatures largely depend on the polymer molecular weight, especially at lower molecular weights. The glass transition temperature increases with molecular weight and approaches a maximum value asymptotically. This effect is not pronounced for high molecular weight polymers. This explains why there is a considerable difference in laser sintering processability between high and low molecular weight polymers [18].

Semi-crystalline polymers usually go through a flow transition and become liquid when the temperature is increased above the melting temperature. Such a transition can be observed in polyethylene with a molecular weight of less than 5 × 105 g/mol. Polyethylene with a molecular weight above 5 × 105 g/mol cannot flow easily as the entanglement of the large polymer chains stops it from flowing. For this reason, UHMWPE does not show a flow transition [17].

The molecular weight is one of the important parameters for describing the polymer characteristics and it affects the quality of laser sintered parts through the melt viscosity. The melting viscosity is directly affected by the weight-average molecular weight (MW) of a linear polymer and this relationship is described in Equation (3). The melt viscosity varies greatly with the change of the molecular weight. The higher molecular weight causes higher melt viscosity [19,20], which can decrease the consolidation of the powder and makes it more difficult to process.

(3) $$\eta_{0}\;=\;k{\left(M_{w}\right)}^{n}$$

where *η*_0_ = zero shear melt viscosity, *k* = constant depends on the material and is sensitive to the density, *n* = 3.4 (if *M_W_* ≥ entanglement molecular weight), and 2.5 ≥ *n* ≥ 1.0 (if *M_W_* ≤ entanglement molecular weight).

## 2. Materials and Methods

The UHMWPE powder used in fabricating the sintered parts, in this study, was UHMWPE GUR 2122 (Celanese, Sulzbach, Germany). UHMWPE GUR 2122 has a molecular weight of around 4.5 × 106 g/mol and a unique morphology with a low powder bulk density of 0.20–0.25 g/cm^3^ (PE-UHMW GUR^®^ 2122 datasheet (Celanese, Sulzbach, Germany).

### 2.1. Particle Size and Morphology

A Mastersizer 3000 (Malvern Instruments, Malvern, UK) was used to determine particle size by laser diffraction. Dry powder dispersion technique, with air as the media, was used to characterise the powder. A feed pressure of 0.3 MPa was used to determine the distribution of the particle size and ten measurements from the available sample were taken.

A scanning electron microscope (Philips XL-20, Philips, Eindhoven, Holland) was used to examine the microstructure of the UHMWPE powder at an accelerating voltage of 10 kV. The sample was prepared for examination by dipping the sample holder, with double sided adhesive tape, in the powder. The sample holder was then shaken up to remove the extra powder and leave a small number of particles. Gold sputtering was used to coat the sample before the examination.

### 2.2. Density of UHMWPE Powder

The true, bulk, and tapped density of UHMWPE powder were measured and the powder flow was investigated.

#### 2.2.1. True Density

The true density is the density of the solid material without the volume of any open and closed pores. The true density can match the theoretical density of the material, depending on the material molecular arrangement. Therefore, it can be indicative of the degree of crystallinity or the ratios of a binary mixture.

Gas pycnometry was used to measure the true density. This uses a gas displacement method to measure volume of materials precisely. Helium is used as the displacement medium [21,22,23]. Helium, with the smallest stable atom, has the capability to fill the inner porosity by diffusion. The specimen is sealed in the compartment of the pycnometer (with known volume), the helium gas is allowed in, and then expanded into a second internal volume. The pressure before and after expansion is measured and used to determine the specimen’s absolute volume [24] and hence the density (i.e., true density of the powder).

#### 2.2.2. Bulk and Tapped Densities and Powder Flow

The determination of bulk and tapped density is a simple method to assess the flow of powders. The achievable laser sintered part density is directly associated with powder density in the part bed, which is linked to the shape of particles and their free flowing behaviour [25].

The powder bulk density is the mass of the powder per unit volume which includes the gaps between particles and the envelope volume of these particles. The powder bulk density can change dramatically, depending on the manner the particles are packed together. It will also change after compaction and consolidation. Therefore, there is not a unique value for density of a particular powder. Two basic methods are widely used for determining the bulk density of a powder; bulk density and tapped density. These densities can provide a guide to the flow characteristics of powders [26].

The bulk density is obtained by pouring a powder into a container and allows it to settle under the effect of gravity. A low-density powder generally has a high powder structural strength and does not collapse easily when poured in a container. On the other hand, a high bulk density powder is structurally weak and collapses easily. This behaviour depends on the friction between the particles and the particle shape. The bulk density is low when the friction between particles is high, and vice versa [26].

The tapped bulk density is determined by tapping the container holding the powder. A cohesive powder collapses considerably by tapping, while a weak or free flowing powder exhibits insignificant change. The powder particles lose contact with each other during tapping, resulting in less friction between these particles and allow them to rearrange. Therefore, tapping can improve the packing conditions of the powder [26]. Clearly, the more a powder is tapped and vibrated, the more it consolidates. Therefore, tapping is usually performed repetitively until the change in volume is small between successive tapping runs.

The ratio between the tapped density and bulk density is known as ‘Hausner Ratio’ (HR), and it classifies the flow of powders. The powder classification regarding HR is [27]

- HR < 1.25 high powder flow,
- 1.25 < HR < 1.4 reduced powder flow,
- HR > 1.4 cohesive powder.

Laser sintering powders need to be under a limit of 1.25 (i.e., high flow) to be considered as a suitable powder with an adequate density [27]. Powder flow and packing influence the layer density of the powder in powder bed. When the powder flows easily and better, the interaction between the particles is weak, resulting in fewer voids and thus a high density. The layer density is directly related to the laser sintering process. A high layer density decreases porosity and improves the part quality and accuracy [28].

HR can be affected by particle shape (i.e., irregular, spherical and flake) and particle size. As particle shape deviates from spherical, then the HR becomes higher. Also, the HR decreases with increasing particle size [26].

#### 2.2.3. Methodology

**Figure 2:**
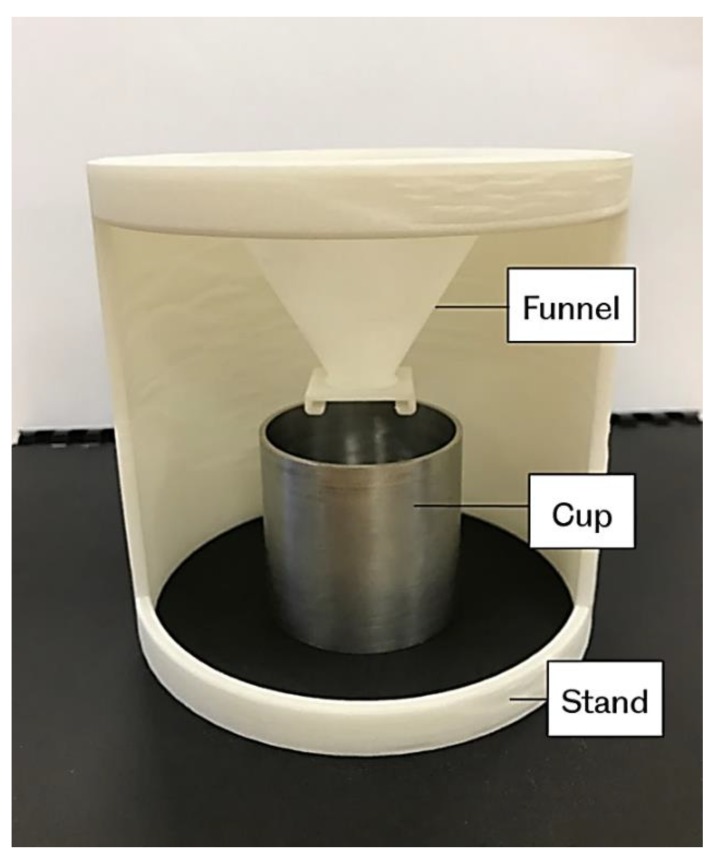
Bulk density measurement set-up.

**Figure 3:**
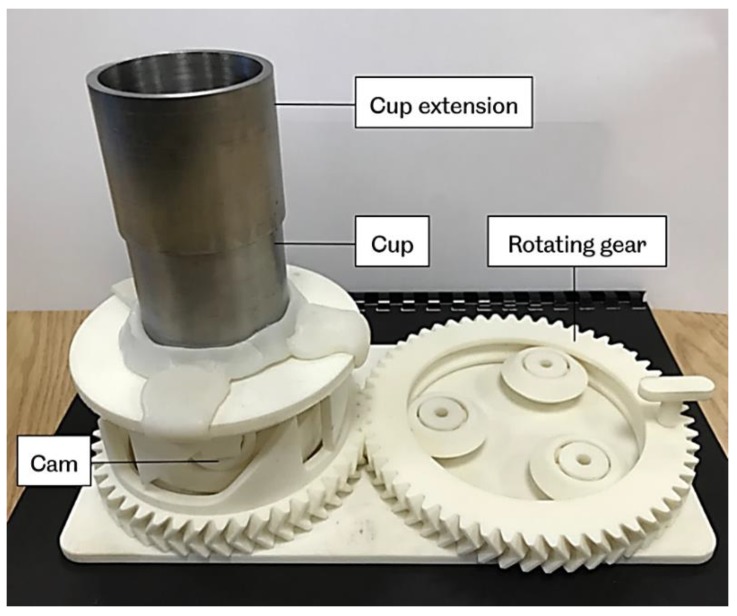
Tapped density measurement set-up.

The bulk and tapped densities measurement set-up are shown in Figure 2 and Figure 3, respectively. The powder bulk density is determined by pouring powder through a sieve in to a pre-weighed 100 cm^3^ steel cup. To simulate the powder feeding process in laser sintering, the powder flowed through a funnel before reaching the cup. The height of the funnel is 60 mm with an orifice of 9 mm diameter. The excess powder is removed carefully from the top of the cup by a metal ruler without affecting or compressing the settled powder.

For the tapped density, a cup extension was placed on the cup containing the initial powder. More powder was then added into the extension cup. Then the entire arrangement was subjected to tapping by using a tapping mechanism as shown in Figure 3. The tapping action is delivered by a rotating cam, which is driven by gears operating manually. The cam lifts the cylinder platform by 13 mm and then drops again. A recommended number of 500 taps was used over a period of 4 minutes. During the tapping process, care was needed to make sure that the powder’s level does not fall below the top edge of the bottom cup, by adding extra powder to the cup extension. After completing the tapping process, the extension cup was removed, and the extra powder was then removed as previously. The content of the cup was weighed, and the tapped density was calculated. These experiments were performed at room temperature.

The experiment was repeated three times on each sample for bulk and tapped densities. Three estimates of Hausner ratio were calculated and the average values were taken.

### 2.3. Differential Scanning Calorimetry (DSC)

Differential scanning calorimetry (DSC) scans were performed (DSC 8500, PerkinElmer, Waltham, MA, USA) to determine the melting and crystallisation parameters. Heating and cooling cycles were used at a temperature rate of 10 °C per minute in a nitrogen atmosphere. The start and end temperature of 25 °C and 220 °C, respectively, was used with a hold of 1 minute at 220 °C before the cooling cycle started. The results were then analysed using the Pyris™ software (PerkinElmer, Waltham, MA, USA). The melting and crystallisation peaks were clearly identified.

### 2.4. Thermogravimetric Analysis (TGA)

Materials for laser sintering must withstand a high thermal load during the preheating stage and also the laser application. Therefore, determining the thermal stability of the polymer is highly recommended.

TGA is a common method of evaluating degradation of materials and is used to identify the various thermal transformations of the material during sintering. TGA is a thermal analysis that determines mass changes as a function of time and temperature in a controlled atmosphere using a highly sensitive microbalance. The technique is useful for compositional analysis of multi-component material or blends, thermal stability, moisture and volatiles content [29].

TGA was undertaken (Pyris 1 TGA, PerkinElmer, Waltham, MA, USA) to investigate the thermal stability of UHMWPE. Approximately, 10 milligrams of material were placed in a ceramic crucible and heated from 25 °C to 600 °C under nitrogen atmosphere at a heating rate of 10 °C /min.

### 2.5. X-Ray Diffraction (XRD) Analysis

X-ray diffraction (XRD) analysis of the polymer provides details regarding crystallinity, size, and orientation of crystallite and phase composition in semi-crystalline polymers [30].

The XRD pattern of a completely crystalline material is a set of sharp peaks, each representing a reflection from one of the various crystallographic planes. A completely amorphous material shows an XRD pattern with a broad peak that represent the average separation of the atoms along and between the polymer chains. Therefore, an XRD pattern of a semi-crystalline polymer is a superposition of the reflections of the crystalline on an amorphous halo. An index of crystallinity can be obtained from the ratio of the crystalline peaks integrated intensity to the total area under the XRD curve [30].

XRD analysis was carried out using a Bruker D8 Advance X-ray diffraction system, equipped with a Cu Kα radiation source with a wavelength of *λ* =  1.5418 nm generated at 40 kV and 40 mA. UHMWPE powder samples were mounted on the stage and scanned from 10° to 70° using a step size of 0.05° and time per step of 10 seconds.

### 2.6. Degree of Crystallinity

It is commonly known that many of the chemical and mechanical properties (such as Young modulus, yield stress, strength, fatigue, and shrinkage) of polymers are affected by the degree of crystallinity [31,32,33,34]. The degree of crystallinity in polymers can be determined by various methods, such as thermal analysis using DSC, volumetric analysis, infrared, Raman spectra, and X-ray diffraction. Each of these methods does not produce identical results, mainly because each method measures different physical parameters and morphological structures [35]. In this work, DSC and XRD methods were used to determine the degree of crystallinity of UHMWPE powder.

Differential Scanning Calorimetry: is used to determine the overall degree of crystallinity of UHMWPE. The degree of crystallinity (XC) was calculated as Equation (4):

(4) $$X_{C}\left(\%\right)=\left(\frac{\Delta H}{\Delta H_{100}}\right)\times 100$$

where *∆H* is the heat of fusion and *∆H*_100_ = 293 J/g is the melting enthalpy of polyethylene at 100% crystallinity [36].

The average of three DSC measurements was used to determine the overall crystallinity of the sample.

X-ray diffraction is a widely used technique to determine the degree of crystallinity in polymers. The diffraction profile is divided in two parts—sharp peaks represent the diffraction of crystallites, and the broad peaks represent the scattering of amorphous phase.

The intensity of the scattering can be measured and used to calculate the degree of crystallinity using the following Equation (5) [37]:

(5) $$X_{C}\left(\%\right)=\frac{I_{C}}{I_{C}+I_{A\;}}\;\times 100$$

where *I_C_* and *I_A_* are the scattered intensities for the crystalline and the amorphous phases, respectively.

Since the assumption is that the areas under the peaks are proportional to the scattering intensities of both crystalline and amorphous phases, so the degree of crystallinity was calculated using the following Equation (6) [30,37,38]:

(6) $$X_{C}\left(\%\right)=\frac{A_{C}}{A_{C}+A_{A\;}}\times 100$$

where *A_C_* represents the area of crystalline phase (i.e., *A*_110_ + *A*_200_) and *A_A_* is the area of amorphous phase.

### 2.7. Hot Stage Microscopy

The consolidation process in laser sintering is initiated by heat, generated by a laser, which results in the formation of the part layers. The Frenkel theory states that the sintering process begins with the formation of necks between adjacent particles, which is then followed by full coalescence. The sintering behaviour can be predicted by direct visualisation of the coalescence on a hot stage microscope [10,11,39].

The simulation of the sintering process is an important stage for the development of new materials in polymer processing [16]. Therefore, Hot Stage Microscopy (HSM) procedure will aid to visually record the event of consolidation of UHMWPE powder and practically verify its consolidation behaviour.

The optical hot stage experiments were performed using an Olympus BX50 optical microscope (Olympus, Tokyo, Japan) equipped with a Linkam heating stage (Linkam Scientific Instruments, Surrey, UK). The temperature was controlled by a Linkam PR600 thermal controller (Linkam Scientific Instruments, Surrey, UK). Images were captured by a VisiCam 10.0 video camera (VWR International, Leuven, Belgium) and were analysed using VisiCam^®^ analysis software (VWR International, Leuven, Belgium). The microscope was fitted with crossed polarising filter, which was used to assess whether spherulites were present within the UHMWPE.

The powder samples were spread sparsely onto glass cover slide and the slide was then placed in the hot stage sample holder. The maximum temperature limit was set to 230 °C, with a heating rate of 10 °C per minute. The camera starts capturing the images when the stage reached 50 °C and once the stage reached the maximum temperature the heating was stopped, and the stage was left to cool back to room temperature. Then the images of the fully melted powder and the development of crystallisation, followed on subsequent cooling to the crystallisation temperature and room temperature, were analysed.

## 3. Results and Discussion

### 3.1. Particle Size and Morphology

**Figure 4:**
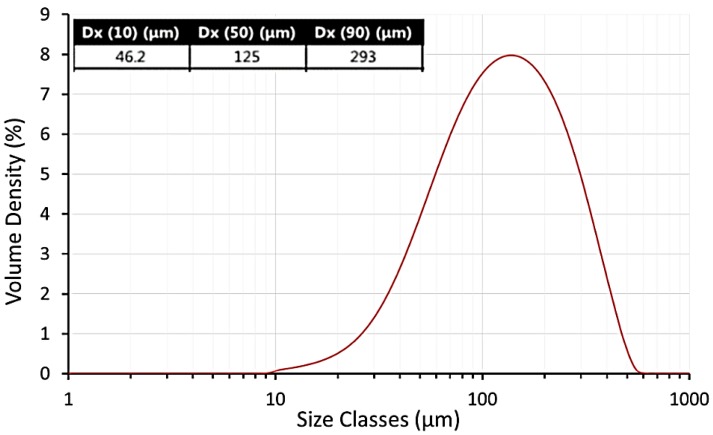
Particle size distribution of ultra-high molecular weight polyethylene (UHMWPE GUR 2122) powder.

Figure 4 shows that the average particle size of UHMWPE used in this work was 125 μm. This result indicates that the average particle size of UHMWE is larger than that of the standard powders used in laser sintering. The commercial powders consist of spherical particles with an average particle size of 60 µm and a narrow size distribution. These powders have a good powder flow properties that are desirable for laser sintering [40].

**Figure 5:**
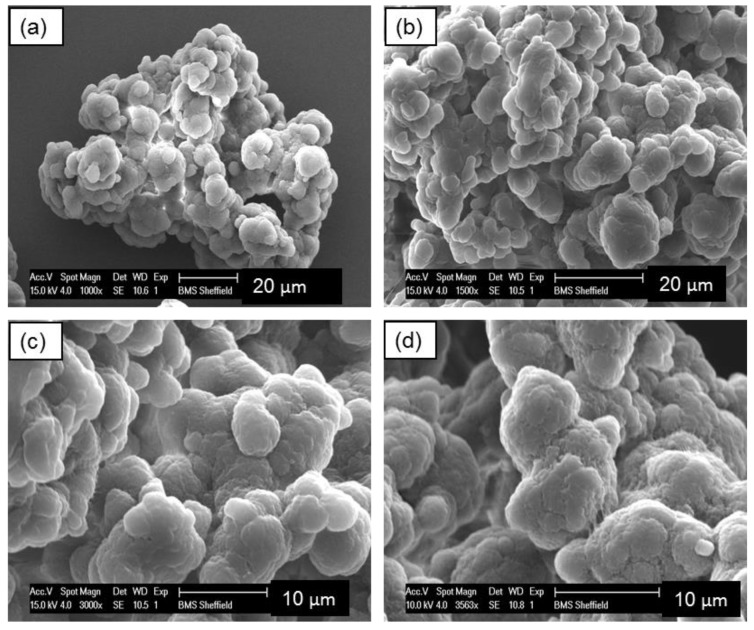
Scanning electron microscopy (SEM) images of virgin UHMWPE powder. The image shows the irregular structure and non-spherical shape. Magnifications: (**a**) 1000×; (**b**) 1500×; (**c**) 3000× and (**d**) 3500×.

SEM examination reveals that the particles of UHMWPE powder are non-spherical in shape with a highly agglomerated structure of smaller particles, as shown in Figure 5. Fibrils also exist in the microstructure of the UHMWPE powders.

UHMWPE particle microstructure is highly complex and from the SEM seems porous. Therefore, the surface structure reduces the ability of the material to pack into regular structures [5].

### 3.2. Density of UHMWPE Powder

The true density of UHMWPE powder was determined by using a helium gas pycnometer (Micromeritics AccuPyc II 1340, Norcross, GA, USA) and was found to be 0.954 g/cm^3^. The bulk density, tapped density, and Hausner ratio for UHMWPE used in this work are shown in Table 1.

Based on the HR criteria (Section 2.2.2) of distinguishing between cohesive and non-cohesive powder, the Hausner ratio of UHMWPE (GUR 2122) powder was found to be just below 1.4. This result indicates that the powder has decreased flow and is cohesive. This behaviour became evident during spreading and distribution of the powder in the laser sintering machine.

The shape and surface of the powder particles determines the behaviour of the powder [25]. The irregular shape leads to poor powder flow, which in turn leads to poor powder deposition and subsequent LS processing difficulties [41]. As discussed in Section 3.1, SEM examination reveals that the particles of UHMWPE powder are non-spherical in shape with a highly agglomerated structure of smaller particles with fibrils also exist in the microstructure of the UHMWPE powders. Angular and point shaped particles show irregular particle formulations within a bulk structure. The irregular rough surface morphology of the particles result in interparticle bridges and internal friction forces [42]. Ideally, the particles of laser sintering powders should be approximately spherical to induce a free flowing behaviour and ensure the powder is distributed on the part bed, by a blade or a roller and will not be compacted further [25].

### 3.3. Differential Scanning Calorimetry

**Figure 6:**
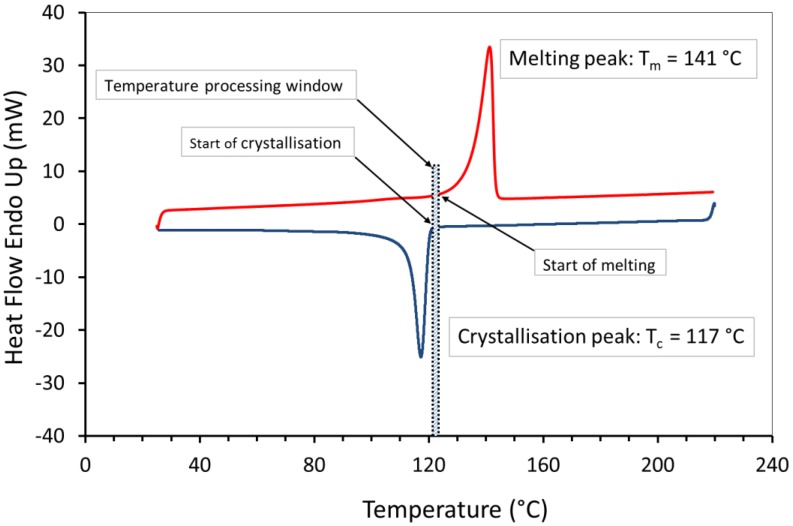
DSC curve of first heating cycle of UHMWPE powder (GUR 2122). The sample was heated at 10 °C per minute from 25 to 220 °C with a hold of 1 minute at 220 °C. The dotted lines show the narrow processing window of 5 °C.

Figure 6 shows very clear distinction between the thermal transitions with a sharp melting point, which is due to the highly ordered crystalline regions in the polymer chains. The melting (Tm) and the peak of crystallisation (Tc) temperature of the UHMWPE were around 141 °C and 117 °C, respectively. These temperatures were used to determine the required build temperature for the powder bed (i.e., processing window). The UHMWPE processing window, which is the difference between the onset melting temperature and the onset crystallisation temperature [43], appears to be narrow (*ΔT* < 5 °C), and this could cause a substantial problem if there are small fluctuations in bed temperature or laser power. However, a part bed temperature was defined by observing the powder flow across the powder bed as it was spread by the blade. Preliminary experiments with UHMWPE, observed that the parts curl and the build fails if precise process parameters, i.e., laser power combined with correct bed and feed temperatures, were not implemented [6].

### 3.4. Influence of Cooling Rate on Processing Window

Laser sintering is a slow process, in which the temperature of the powder bed is held close to the melting point for a long time, and new pre-heated layers are spread on top of the previously sintered layers [40]. In this work, an attempt was made to investigate the melting and crystallisation behaviour of UHMWPE and simulate LS process by DSC measurements. Using different cooling rates clearly shows that crystallisation has a high time dependency. Therefore, several heating and cooling rates (1, 5, and 10 °C/min) were used (Table 2) and the resulting data were analysed. The previous thermal history of the virgin powder was first cleared by the first heating and cooling cycles and therefore these cycles were not considered in this analysis. Note that, due to restrictions on capability of analysis equipment, the heating/cooling rates that we have employed are, necessarily, many times slower than those in Laser Sintering.

**Figure 7:**
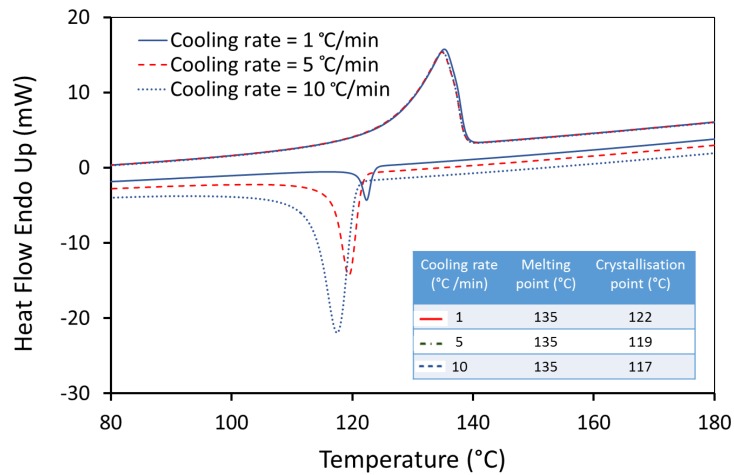
DSC curves of UHMWPE at different cooling rate. The same sample was heated from 25 °C to 220 °C at 10/min.

Figure 7 shows that the crystallisation starts at a higher temperature with lower super-cooling of the molten UHMWPE material as the cooling rate is reduced. A considerable change in the temperature of the crystallisation was observed with a difference of 5 °C between a cooling rate of 1 °C/min and 10 °C/min. The sintering of UHMWPE can be influenced significantly, as the difference between melting and crystallisation peaks is narrow at lower cooling rates. In addition, a significant change in the width of the peaks during the control cooling steps was observed. The peak width increases with the increase in cooling rate, suggesting an increase in the distribution of crystal lamella size [44].

### 3.5. Thermogravimetric Analysis

**Figure 8:**
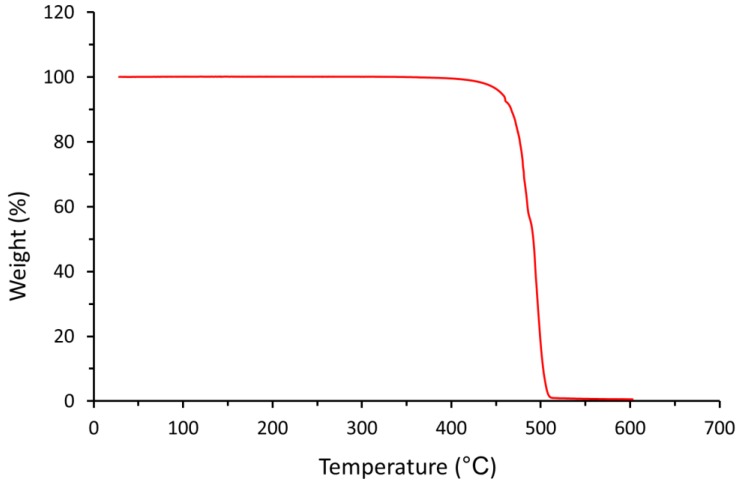
TGA analysis of UHMWPE. Showing the start and the end onset temperatures of degradation at 478 °C and 513 °C, respectively.

The TGA analysis reveals a stable weight up to about 400 °C, as shown in Figure 8. At higher temperature, thermal degradation of the UHMWPE begins with the onset appearing at approximately 478 °C. Therefore, UHMWPE should be sufficiently stable, and degradation should not occur during the sintering process. The result indicates that no trace or a release of any significant volatile solvent residue that can be detected but a limited amount of residue remaining at 513 °C (1.0%) was observed.

### 3.6. X-Ray Diffraction Analysis

**Figure 9:**
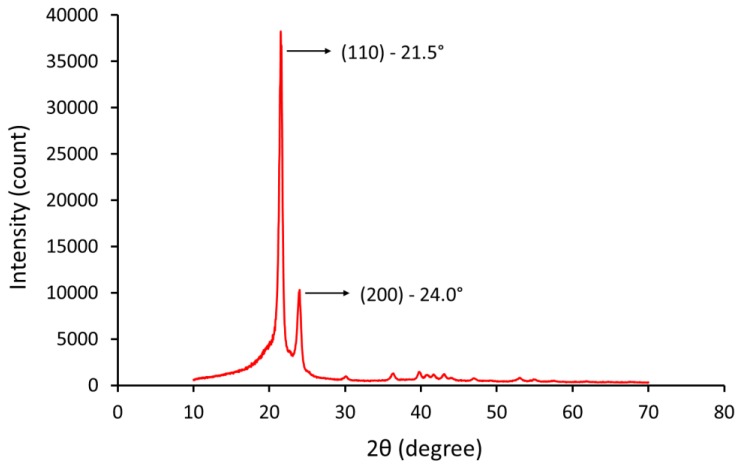
X-ray diffraction pattern of UHMWPE powder.

The XRD spectrum for UHMWPE powder is shown in Figure 9. The result shows that UHMWPE is a semi-crystalline polymer which consists of two phases; an amorphous phase and the expected orthorhombic crystalline phase of polyethylene with two distinctive sharp peaks at 2θ = 21.5° (110) and at 2θ = 24° (200) [45].

### 3.7. Degree of Crystallinity

The degree of crystallinity of UHMWPE powder was determined using DSC and XRD methods and the result is shown in Table 3.

The results show that the crystallinity value obtained by XRD is higher compared to DSC measurements. This variation was expected since the crystallinity obtained via DSC analysis is measured under a given thermal profile (i.e., dynamic measurement), where the crystallinity obtained via the XRD analysis is measured under a constant temperature.

### 3.8. Effect of Cooling Rate on Crystallisation

**Figure 10:**
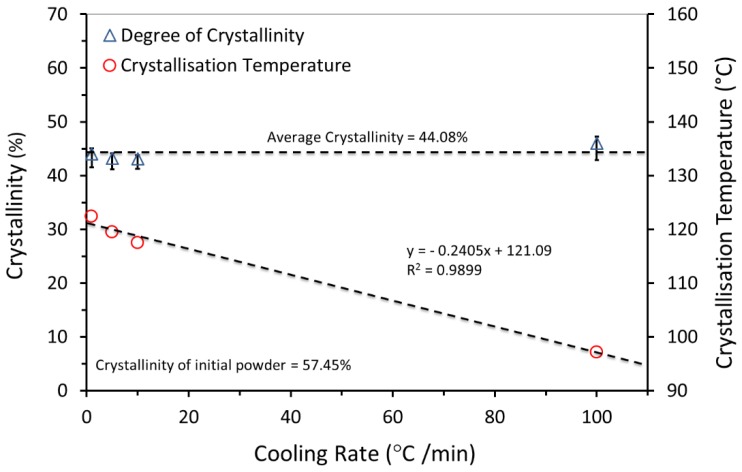
Influence of cooling rate on the crystallisation of the UHMWPE.

Referring to Section 3.4, the laser sintering process was simulated by DSC to investigate the effect of cooling rate on crystallisation behaviour. Cooling rates of 1, 5, and 10 °C/min were used, as listed in Table 2, with an additional cycle with cooling rate of 100 °C/min. The resulting data were presented in Figure 10.

The result in Figure 10 shows that with the increase in the cooling rate, the crystallisation temperature is decreased. This indicates that the crystallisation was faster for higher cooling rates, implying the nucleation-dominated process [44]. A slower cooling rate causes the crystallisation of the UHMWPE matrix to be slower (i.e., more perfect crystals). Literature suggests that the final crystallinity of UHMWPE is improved [46]. However, during these measurements, different cooling rates did not alter the degree of crystallinity of UHMWPE significantly, as can be seen by the equal areas of the subsequent melting peaks. This behaviour is related to the extremely long molecular chains in UHMWPE.

### 3.9. Effect of Heating on Crystallisation

Many factors significantly affect the properties of laser sintered parts, such as laser energy and powder bed temperature, which influence the degree of crystallinity [47]. Crystallinity of polymers depends on the thermal history, which is a widely observed behaviour of semi-crystalline thermoplastics.

Samples were heated to 117.5 °C, at heating rate of 10 °C/min and held there for 15 min. The heating then continued to 220 °C, and then samples were cooled to 25 °C at 10 °C/min. The same procedure was repeated for the second and third cycles with the first heating temperature set to 120 °C and 125 °C respectively. This protocol melts the more highly folded chain crystals that are created on the cooling step. Thus at 117.5 °C highly folded polymer crystals melt and reorganise to become less chain folded. Table 4 shows the DSC experiment protocol.

**Figure 11:**
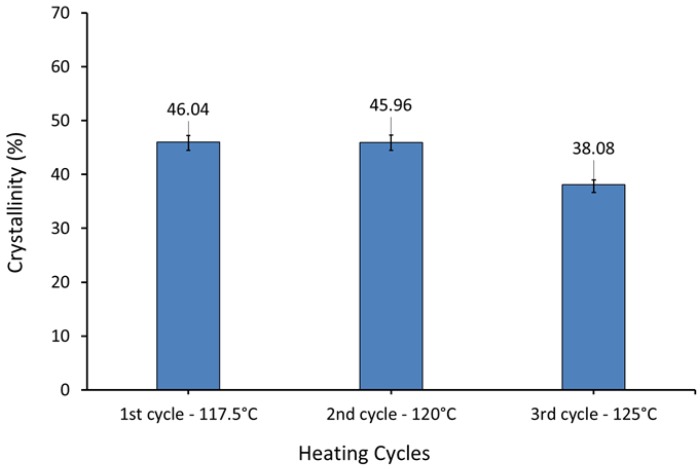
Influence of heating on the crystallisation of the UHMWPE (DSC measurements).

Figure 11 shows that the degree of crystallinity of UHMWPE increased with increased super-cooling.

In laser sintering, the energy input comprises two components—the first is due to the bed temperature, the second due to the laser. The part bed is preheated and kept at a constant temperature (i.e., part bed temperature) during the process. However, the temperature locally increases when the laser is selectively applied to heat the powder above the melt temperature. Therefore, a higher bed temperature results in increased energy input, and this impacts on the properties of the parts produced.

### 3.10. Hot Stage Microscopy

**Figure 12:**
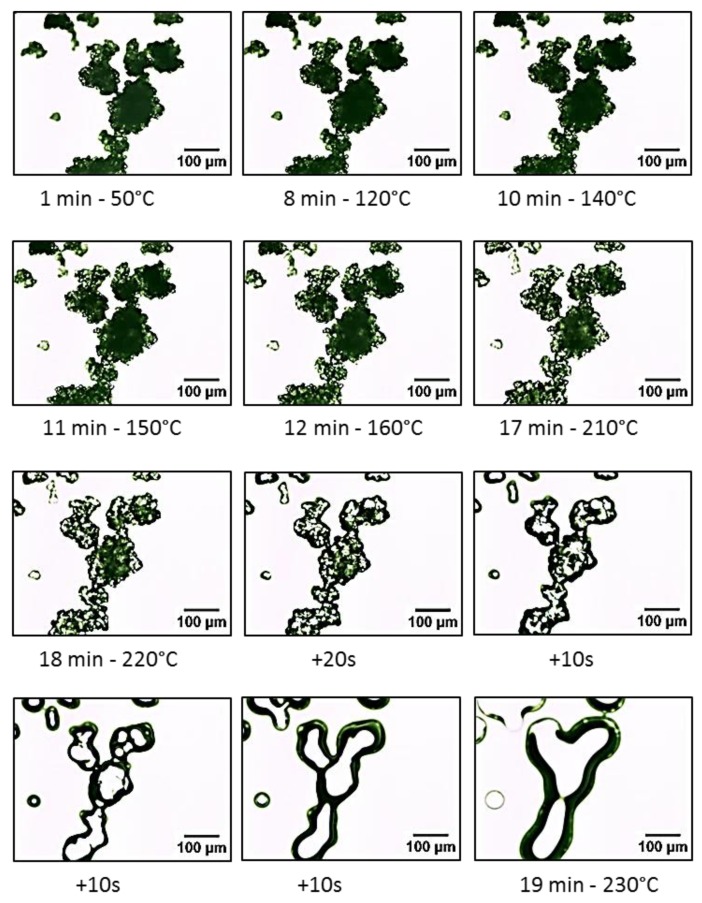
Hot stage microscopy images of UHMWPE.

The optical images of the UHMWPE particles during coalescence are presented in Figure 12. A series of time lapse images of a number of UHMWPE particles sintering together on a hot stage.

The images suggest that the particle morphology of UHMWPE did not change, and the particles preserved their confirmations even after the hot stage reached the melting temperature of UHMWPE (i.e., 141 °C). Andjelić et al. suggested that the chain location remains un-changed, even after melting. They also suggested that the UHMWPE lamellae contains a high concentration of entangled tie macromolecules in the amorphous region. The formation of these stable entanglements will prevent significant diffusion of the chains [44].

Noticeable changes were observed at temperature above 160 °C and as the sintering progresses the particles coalesced completely and become translucent as the crystalline phase becomes amorphous.

To confirm the above observation, a DSC test was carried out on a sample of powder with an open pan. The start temperature of 25 °C and an end temperature of 150 °C were used with heating and cooling rates of 10 °C/min.

**Figure 13:**
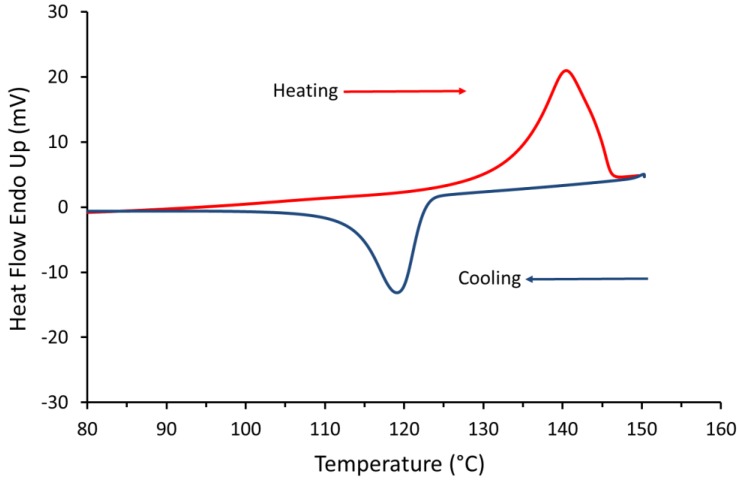
DSC curve of UHMWPE powder heated to 150 °C.

The result of the test, shown in Figure 13, clearly confirms that the surface of the UHMWPE powder was melted when the temperature reached 150 °C.

After the DSC test was completed the powder pan was examined visually. The powder resembled a frozen powder without any signs of flow and the irregular structures of the powder were preserved.

**Figure 14:**
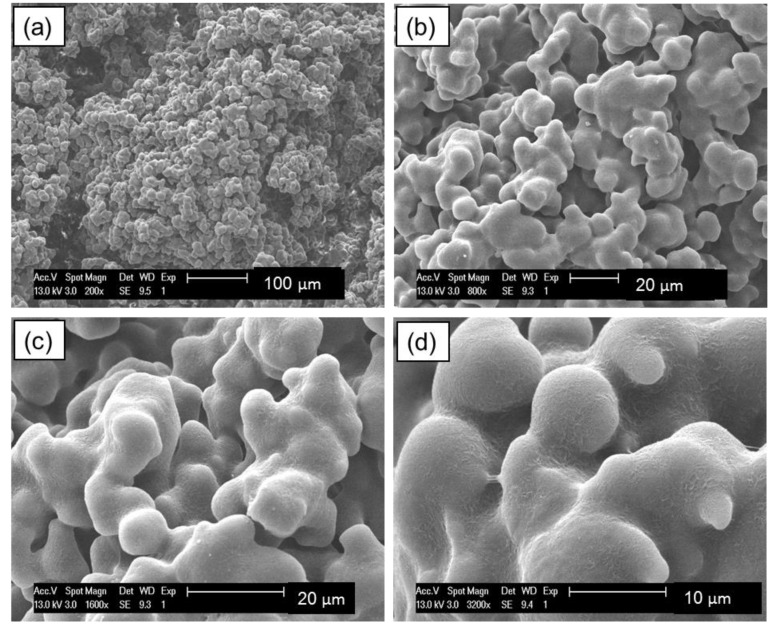
SEM images of UHMWPE powder heated to 150 °C and cooled down to room temperature. Magnifications: (**a**) 200×; (**b**) 800×; (**c**) 1600× and (**d**) 3200×.

The same sample was then examined under the SEM and the result is shown in Figure 14. The images clearly show that the particles are indeed coalesced, and fusion was evident at 150 °C. These results confirm the findings form the hot stage microscopy test.

The sintering of UHMWPE is not governed by the viscous flow, but it is affected by its unique particle morphology. Therefore, the Frenkel-Eshelby model would underestimate the sintering rate of UHMWPE and leads to a substantial deviations from the experimental result [11].

**Figure 15:**
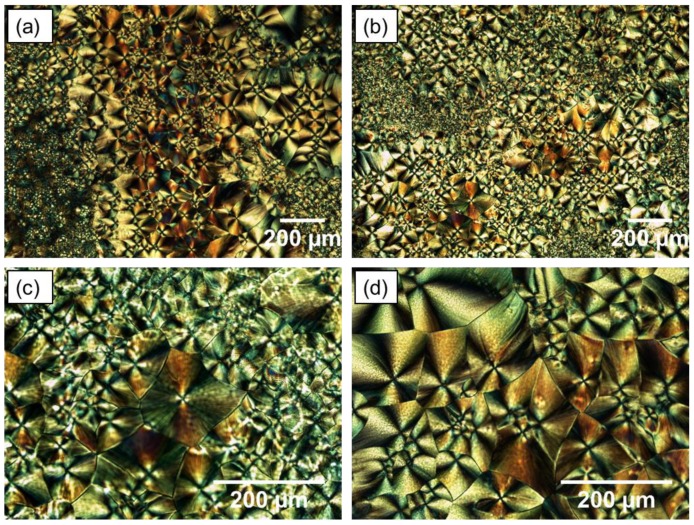
Polarised light microscopy images of UHMWPE. Magnification: (**a**,**b**) = 4×; (**c**,**d**) = 10×.

The development of crystallisation within the UHMWPE was followed using a polarising light filter. Hot stage images captured during the crystallisation of UHMWPE are shown in Figure 15. A very characteristic pattern was observed, which consists of a large number and well-defined banded of spherulites. Maltese cross-pattern of crystalline regions, small and large spherulites, are clearly defined in UHMWPE structure.

Spherulites are formed from distinct nuclei during solidification. When the undercooling is increased below the melting point, the number of these nuclei is increased. Therefore, when semi-crystalline polymers are cooled quickly, a large number of smaller spherulites are formed. On the other hand, larger spherulites are formed due to slow cooling [31]. The size of spherulites affects the strength and ductility of the polymer. Semi-crystalline polymers containing small spherulites are normally tougher than those consisting coarse spherulites, which have weak boundaries. It was also suggested that an increase in crystallinity or spherulites size reduces the toughness [48].

### 3.11. Laser Sintering Trials of UHMWPE

A commercial laser sintering machine (FORMIGA P100, EOS, Munich, Germany) was used to manufacture test samples from UHMWPE (GUR 2122) powder. Virgin powder was used for all builds.

A range of processing parameters were trialled in order to obtain suitable parameters for processing UHMWPE parts. In the initial trials carried out by the same authors Khalil et al. [49], combinations of laser powers and laser scan speeds with a range of powder bed temperatures, removal chamber temperatures, laser counts and hatch spacing were attempted (Table 5). It was found that a bed temperature and removal chamber temperature of 142 °C and 135 °C, respectively, were suitable to achieve acceptable spreading and multilayer sintering of UHMWPE powder.

However, a couple of issues were observed including unease of cleaning and powder removal of the laser sintered parts. Therefore, Khalil et al. attempted to used low levels of laser powers (i.e., 6, 8, 10, and 12 watts) which then found to be suitable for powder spreading and reducing the effect of curling during laser sintering as well as the ease of cleaning and powder removal in post processing.

**Figure 16:**
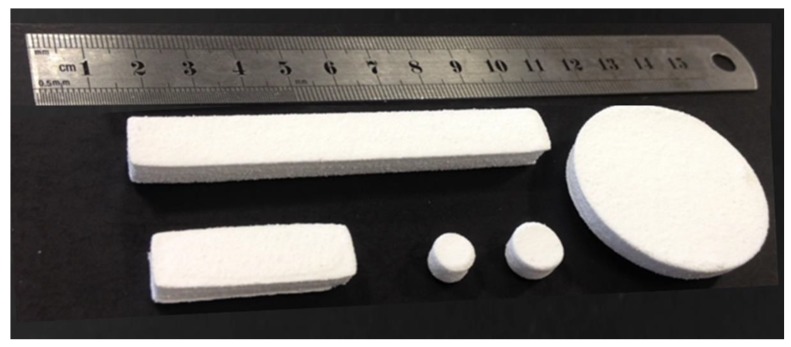
UHMWPE parts produced by laser sintering (LS) machine EOS P100.

Processing UHMWPE GUR 2122 by laser sintering was challenging, however, LS parts were manufactured successfully at various laser power [49,50]. Figure 16 shows different UHMWPE parts were produced with a good definition and significant structural integrity for the parts to be handled.

## 4. Conclusions

The particles of UHMWPE powder are non-spherical in shape, with a highly agglomerated structure of smaller particles with fibrils also exist in the microstructure of the UHMWPE powders. The particles of laser sintering powders should be approximately spherical in order to induce a free flowing behaviour. The Hausner ratio of UHMWPE powder was found to be just below 1.4 indicating that the powder has decreased flow and is close to being cohesive which is not favourable for laser sintering.

DSC result shows that UHMWPE has sharp thermal transitions indicating areas of crystallinity. Above the melting temperature, UHMWPE has transformed, and a neck growth occurred. However, UHMWPE requires a longer exposure before the neck formation and coalescence of the particles. This behaviour was confirmed by the hot stage microscopy. The DSC curve shows that the processing window of UHMWPE is narrow (less than 5 °C) and this could cause a substantial problem during laser sintering process if small fluctuations in bed temperature or laser power occurs.

In laser sintering, the laser provides energy to melt the polymer, but applying high energy could damage and degrade the polymer chain. TGA analysis was used to examine the degradation of UHMWPE and assess the window between the melting and degradation of UHMWPE. The result clearly shows that the temperature window is wide enough and favourable for laser sintering in terms of material degradation.

It is commonly known that many of the chemical and mechanical properties of polymers are affected by the degree of crystallinity, such as Young modulus, yield stress, fatigue, and shrinkage. X-ray diffraction spectroscopy was used in this study to verify measurement of the degree of crystallinity with DSC. The degree of crystallinity found to be 57.45 ± 3.70 and 67.80 ± 8.50 as measured by DSC and XRD, respectively. The result shows that the crystallinity value obtained by XRD is higher compared to DSC measurements.

The effect of cooling rate on the temperature and degree of crystallinity was investigated. The result shows that with the increase in the cooling rate, the crystallisation temperature is decreased. However, different cooling rates did not alter the degree of crystallinity of UHMWPE significantly. This behaviour is very much affected by the extremely long molecular chains in UHMWPE.

The sintering behaviour of UHMWPE is not only controlled by the viscous flow, but it is affected by the morphology of the particles.

Although, the commercial UHMWPE GUR 2122 powder was successfully sintered, the low density, poor sphericity, irregular rough surface morphology, agglomeration, and variable microstructure make this powder far from ideal for laser sintering. Enhancing the density, particle size, and shape of the UHMWPE powder would improve the flow properties of the powder and then induce a better packing powder. Furthermore, polymer modification to achieve a wider thermal processing window may increase the feasibility of processing the UHMWPE.

## Figures and Tables

**Table 1:** Densities and Hausner ratio of UHMWPE powder.

Bulk Density (g/cm^3^)	Tapped Density (g/cm^3^)	Hausner Ratio
0.232 ± 0.002	0.324 ± 0.002	1.390 ± 0.019

**Table 2:** DSC experiment protocol to examine the effect of cooling rate on processing window.

Cycle No.	Start (°C)	Dwell (min)	Heating Rate (°C/min)	End (°C)	Dwell (min)	Cooling Rate (°C)	Final (°C)
1	25		10	220	1	10	25
2	25	1	10	220	1	10	25
3	25	1	10	220	1	5	25
4	25	1	10	220	1	1	25

**Table 3:** Degree of crystallinity (X_C_) of UHMWPE powder.

Method	Crystallinity (%)
DSC ^1^	57.45 ± 3.70
XRD	67.80 ± 8.50

^1^ Crystallinity was measured from the first heating cycle.

**Table 4:** DSC experiment protocol.

Cycle No.	1st Heating (°C)	Hold Time (min)	2nd Heating (°C)	Dwell (min)	Cooling (°C)
1	25	1	10	1	25
2	117.5	15	10	1	25
3	120	15	10	1	25
4	125	15	10	1	25

**Table 5:** Process parameters used in the initial trials of laser sintering of UHMWPE.

Parameter	Unit	Range
Laser power	watt	13, 17, 18, 23 and 29
Laser scan speed	mm/s	1500 and 2500
Bed temperature	°C	130 to 145 at five degrees intervals
Removal chamber temperature	°C	125, 130 and 135
Laser count	-	1 (single scan) and 2 (double scan)
Hatch spacing	mm	0.15 and 0.25
